# Soft-Matter Physics Provides New Insights on Myocardial Architecture: Automatic and Quantitative Identification of Topological Defects in the Trabecular Myocardium

**DOI:** 10.3390/jcdd11010011

**Published:** 2023-12-29

**Authors:** Johanne Auriau, Yves Usson, Pierre-Simon Jouk

**Affiliations:** 1 Equipe Biologie Computationnelle et Modélisation, University Grenoble Alpes, CNRS, UMR 5525, VetAgroSup, Grenoble INP, CHU Grenoble Alpes, TIMC, 38000 Grenoble, France; yves.usson@univ-grenoble-alpes.fr (Y.U.); psjouk@chu-grenoble.fr (P.-S.J.); 2 Service de Cardiologie, CHU Grenoble Alpes, CS 10217, CEDEX 9, 38043 Grenoble, France; 3 Service de Génétique, Génomique et Procréation, CHU Grenoble Alpes, CS 10217, CEDEX 9, 38043 Grenoble, France

**Keywords:** topology, disclination, cardiac myoarchitecture, liquid crystals

## Abstract

This article is the third in our series dedicated to the analysis of cardiac myoarchitecture as a nematic chiral liquid crystal (NCLC). Previously, we introduced the concept of topological defects (disclinations) and focused on their visual identification inside the compact myocardium. Herein, we investigate these using a mathematical and automated algorithm for the reproducible identification of a larger panel of topological defects throughout the myocardium of 13 perinatal and 11 early infant hearts. This algorithm identified an average of 29 ± 11 topological defects per slice with a 2D topological charge of *m* = +1/2 and an average of 27 ± 10 topological defects per slice with a 2D topological charge of *m* = −1/2. The excess of defects per slice with a 2D topological charge of *m* = +1/2 was statistically significant (*p* < 0.001). There was no significant difference in the distribution of defects with a 2D topological charge of *m* = +1/2 and *m* = −1/2 between perinatal and early infant hearts. These defects were mostly arranged in pairs, as expected in nematics, and located inside the trabecular myocardium. When isolated, defects with a 2D topological charge of *m* = +1/2 were located near the luminal extremity of the trabeculae and those with a 2D topological charge of *m* = −1/2 were located at the anterior and posterior part of the interventricular septum. These findings constitute an advance in the characterization of the deep cardiac myoarchitecture for application in developmental and pathological studies.

## 1. Introduction

We have previously and for the first time demonstrated that, like some living biological tissues [1,2], the myosin myocardial mesh of the human heart is an analogue of an uniaxial nematic chiral liquid crystal (NCLC), opening new perspectives for its description by using tools from the field of crystallography, soft matter physics and mathematics [3]. In the first article of our series devoted to the cardiac myoarchitecture considered as an NCLC we mainly focused on the description of the compact structure of the left ventricle (LV) [3]. In the second, we introduced the concept of topological defects (disclinations) characterized by a 2D topological charge *m* that we observed by the visual identification of two different types in the compact myocardium of the ventricular mass: *m* = +1 at the LV apex and *m* = −1/2 at the anterior and posterior part of the interventricular septum (IVS) [4]. 

However, recent works on active nematics showed that other topological defect patterns exist than those we visually identified [1,5,6,7]. This led us to write a software program that provides a reproducible and automatic extraction of topological defects with various 2D topological charges within the orientation maps obtained from our collection of fetal and neonatal hearts. The aim of this study was to determine if these topological defects would bring new elements to the description of specific myocardial structures, in particular those like trabeculae that are still poorly understood [8]. 

As for our previous articles, this paper was written in order to guide the reader didactically through this analysis combining topology and anatomy, which although being a precise and reproducible approach is more difficult to apprehend for clinicians and anatomists.

## 2. Materials and Methods

### 2.1. Historical Collection of Fetal and Neonatal Hearts Acquired with PLI-MMA

**Figure 1:**
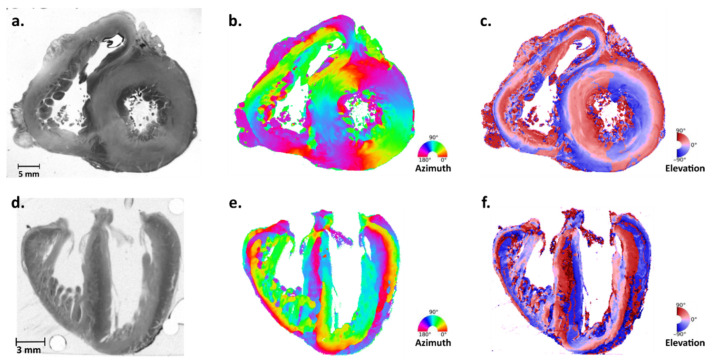
PLI-MMA orientation maps of 500 µm thick sections of two perinatal hearts cut along two orthogonal planes. Upper row: short axis section of a 30-week post-natal heart, seen in simple transmitted light after embedding in MMA resin (**a**). The same section in the color-coded azimuth map with angle ranging from 0° to 180° (**b**) and in the color-coded elevation map with angle ranging from 90° to –90° (**c**). Lower row: transversal section of a perinatal heart (21-week gestational age) seen in simple transmitted light after embedding in MMA resin (**d**). The same section in the color-coded azimuth map (**e**) and in the color-coded elevation map (**f**). Angles are measured relative to the transverse cutting plane.

The Grenoble-Alpes University Hospital has a legally declared collection of embedded heart sections collected after autopsies of fetal, neonatal, and infant deaths performed for diagnostic purposes. The detailed protocol used for histological preparation of heart samples is thoroughly described in our previous publications [4,9]. Briefly, after being fixed, each heart sample was embedded in methyl methacrylate (MMA) and cut into a series of sections spaced every 1000 µm and with a thickness of 500 µm due to an inevitable 500 µm loss of material. Due to the very low crystal birefringence of the myosin molecules, it was necessary to have 500 µm thick sections in order to obtain a maximum phase delay of 250 nm between the ordinary ray and the extraordinary ray. The series extends from the base to the apex (short-axis plane) or, more rarely, from the inferior face (diaphragmatic) to the superior face of the heart (transversal plane). The number of sections varied approximately from 20 to 50, depending on the size of the heart. Then, the mean 3D orientation of all myosin molecules in the voxel was obtained using polarized light imaging (PLI) techniques for each section. This information was organized in a 3D matrix made of 90 × 90 × 500 µm^3^ voxels, each containing the local mean orientation (i.e., the director) of all myosin molecules. As the rod size of the myosin molecule is approximately 1 µm, each voxel contains around 10^8^ myosin molecules. The orientation was expressed by two angles in a Cartesian system with reference to the sectioning plane: the azimuth angle and the elevation angle. False-color maps were calculated to represent the values of the azimuth angles or elevation angles in each section as shown in Figure 1. 

This collection was obtained after obtaining written consent from the parents or guardians at the time of the request for autopsy authorization and for research authorization on normal and abnormal development. The institutional review board of the hospital approved the research protocol and the study was conducted in accordance with the 1964 Declaration of Helsinki and its later amendments. Samples dedicated to research purposes were kept anonymous, but past medical history and patient characteristics were collected when available. For the analysis, we only selected hearts with no cardiac abnormalities. Perinatal hearts are those of patients who died before birth or within the first week of life, and early infant hearts are those of patients who died after the first week of life (>7 days).

### 2.2. Analogy of Myocardial Structure with Nematic Chiral Liquid Crystals (NCLC), Measurement of the 2D Topological Charge

Due to the multiple common physical properties of myosin molecules with NCLC such as their rod-like shape, birefringence and tendency to three-dimensional arrangement along their long axis, we demonstrated that the human myocardium can be considered to be analogous of uniaxial NCLC [3]. As a reminder, in nematics the local “average molecular orientation” is defined by a director field *n*. This director is a 2D-unit vector in which only the orientation is important as molecules have no direction (*n* and −*n* are identical and represent the same physical state). Topological defects or disclinations occur when the order changes discontinuously and takes the form of an isolated point in a 2D space or lines in a 3D space, where the director field is ill-defined. These topological defects are characterized by a topological charge *m* (or winding number) that correspond to the angle that the director turns on a loop around the defect divided by the angle 2π [10,11,12]. This topological charge can be either a positive or negative integer, or a half integer value. Didactical videos of the 2D topological charge calculation process are available in the supplements of our previous article [4].

**Figure 2:**
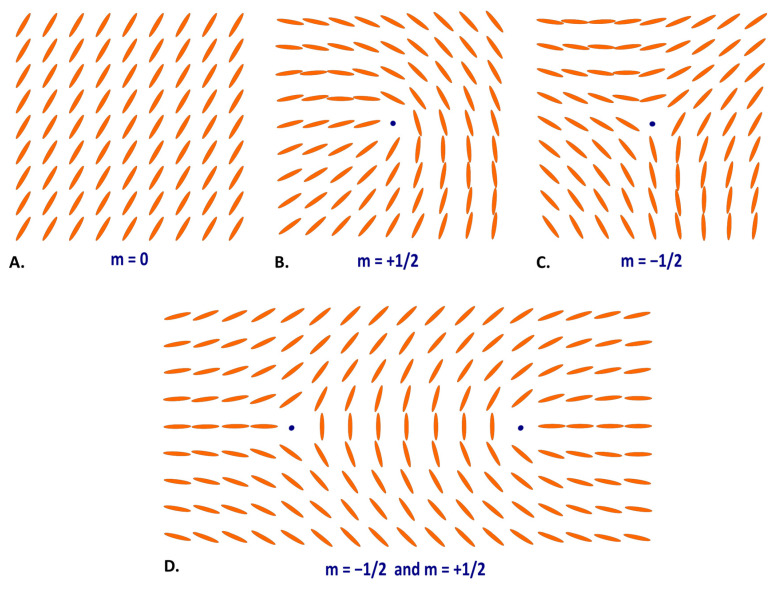
Topological defects as isolated points (blue dots) in a 2D nematic liquid crystal structure. Schematic illustration with rod-shaped molecules organized for different 2D topological charges *m*: (**A**) perfect parallel alignment along their long axis corresponding to a null 2D topological charge *m* = 0, (**B**) comet like pattern with *m* = +1/2, (**C**) threefold pattern with *m* = –1/2. (**D**) Represents the most frequent topological defect pair with *m* = −1/2 and *m* = +1/2.

As a reminder, our material is inert as it is post-mortem myocardial tissue. Thus, we can only describe topological defects constituted during fetal and perinatal life. Figure 2 illustrates various topological defects in a nematic liquid crystal structure with different 2D topological charges *m* that we observed in our material.

In our previous work, we visually identified topological defects with two different 2D topological charges in the compact myocardium of the ventricular mass of 18 perinatal and early infant hearts: a defect with a 2D topological charge of *m* = +1 at the LV apex and defects with a 2D topological charge of *m* = −1/2 at the anterior and posterior part of the IVS [4]. With this visual, subjective, and incomplete identification of topological defects, only large defects could be discerned while smaller ones could be missed. This led us to develop a mathematical algorithm and implement the corresponding software to identify, in a precise and reproducible fashion, defects as isolated points with a 2D topological charge value *m* varying from −1 to +1 with a step of ±1/2 (flowchart of the Algorithm A1 in Appendix A). Topological defect maps were computed from azimuth and elevation data, and false-color-coded according to the 2D topological charge value *m*. Only the center of the defect was represented and superimposed on the streamline maps with a classical line integral convolution (LIC) algorithm that generates a texture image built from limited regional tractographies (5 voxels spans) [13,14]. 

In order to compare and evaluate the changes in distribution of topological defects within and between the different hearts, we calculated the average number per slices of topological defects with the same 2D topological charge *m*. Statistical analyses (Fisher F-test and Student *t*-test) were performed with JASP (Version 0.17.2).

## 3. Results

A total of 13 perinatal and 11 early infant hearts were analyzed. Only one heart of 21 weeks gestational age (GA) was cut in a transverse plane, while the others were cut in a short axis plane and ranged from 29 weeks GA to 20 months after birth. 

**Figure 3:**
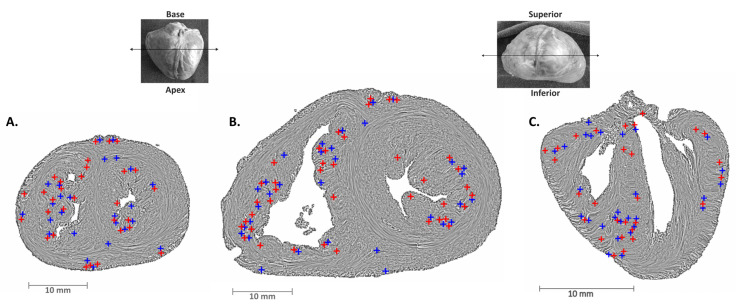
Streamline maps with LIC texture of (**A**) a perinatal heart and (**B**) an early infant heart cut in the short axis plane, and (**C**) of a perinatal heart cut in the transverse plane. The LV is located on the right of the images, and the anterior wall at the top in the short axis sections. Red crosses represent the centers of defects with a 2D topological charge of *m* = +1/2 and blue crosses represent the centers of defects with a 2D topological charge of *m* = −1/2. Upper left thumbnail: upper heart view. The double-headed arrow locates the section plane for panels (**A**,**B**). Upper right thumbnail: view from the apex. The double-headed arrow locates the section plane for panel (**C**).

The centers of the topological defects were superimposed on the streamline maps with LIC texture and false-color-coded according to their 2D topological charge *m* (Figure 3). Videos S1–S3 show examples of topological defects maps where red crosses represent the centers of defects with a 2D topological charge of *m* = +1/2 and blue crosses represent the centers of defects with a 2D topological charge of *m* = −1/2. In Videos S1 and S2, the hearts are cut in a short axis plane from apex to base while in Video S3 the heart is cut in a transverse plane from anterior to posterior. 

The majority of topological defects had a 2D topological charge of *m* = −1/2 or *m* = +1/2. Indeed, no defect with a 2D topological charge of *m* = +1 was found among the entire collection. Only two topological defects with a 2D topological charge of *m* = −1 were found in the RV trabeculae and one in LV trabeculae of early infant hearts.

For the entire collection, the average number per slice of topological defects with a 2D topological charge of *m* = +1/2 was 29 ± 11, and the average number per slice of topological defects with a 2D topological charge of *m* = −1/2 was 27 ± 10.

There were more defects per slices with a 2D topological charge of *m* = +1/2 than *m* = −1/2 in perinatal hearts (27 ± 8 vs. 25 ± 8; *p* < 0.001) and in early infant hearts (33 ± 13 vs. 31 ± 13; *p* < 0.001). 

However, when comparing early infant hearts versus perinatal hearts, there was no statistically significant difference in the average number of defects with a 2D topological charge of *m* = +1/2 (*p* = 0.196). Similarly, there was no statistically significant difference in the average number of defects with a 2D topological charge of *m* = −1/2 (*p* = 0.201).

**Figure 4:**
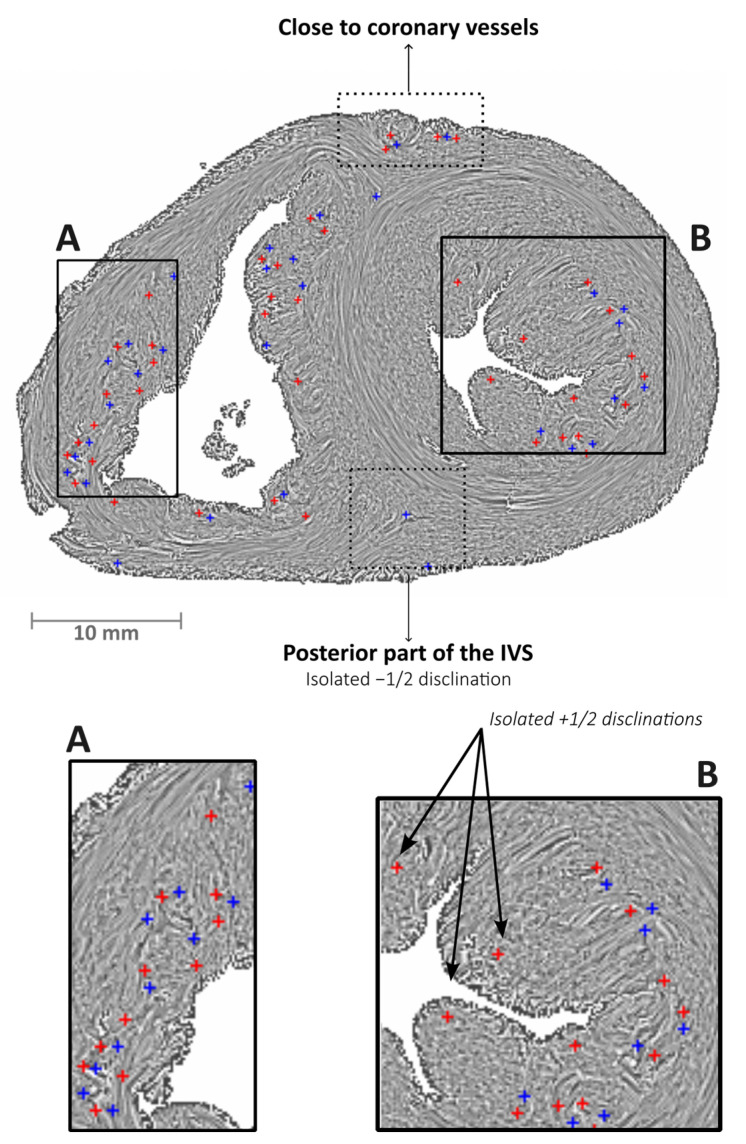
Streamline maps with the LIC texture of an early infant heart in the short axis plane. Red crosses represent the centers of defects with a 2D topological charge of *m* = +1/2 and blue crosses represent the centers of defects with a 2D topological charge of *m* = −1/2. Panel (**A**) shows the free wall of the RV with many pairs of defects with a 2D topological charge of *m* = ±1/2 inside the trabeculae. Panel (**B**) highlights three isolated defects with a 2D topological charge of *m* = +1/2 located near the luminal extremity of the LV trabeculae.

The defects with a 2D topological charge of *m* = ±1/2 were mostly arranged in pairs. Almost all of these pairs were located inside the trabecular myocardium and less frequently near the coronary vessels in the epicardium (Figure 4). When isolated, the defects with a 2D topological charge of *m* = −1/2 were often located at the anterior and posterior part of the IVS, while those with a 2D topological charge of *m* = +1/2 were mainly located near the luminal extremity of the trabeculae (Figure 4B). No pair of defects with a 2D topological charge of *m* = ±1/2 was found in the LV compact myocardium. 

## 4. Discussion

This article is the third in our series devoted to cardiac myoarchitecture considered as an NCLC, and fits into the current effervescent research trend on the analogy of living biological tissues with liquid crystals. Like any living tissue, the myocardium is subject to constant and evolving dynamic constraints, particularly during its development or in pathological conditions. The objectives of this approach are to better characterize and model the internal structure of tissues in order to provide new elements to the understanding of the existing links between biomechanical constraints and cellular processes. The originality of this article is to infer topological concepts issuing from liquid crystals physics to cardiac myoarchitecture.

One of the major findings of this study is the presence of numerous pairs of topological defects with a 2D topological charge of *m* = ± 1/2 inside the LV and RV trabeculae while none were found inside the compact myocardium. Looking more closely, the deepest pairs inside the myocardium were located at the interface between the trabecular and the compact myocardium, also called the trabeculata–compacta interface (TCI) [8]. At this interface, the spatial organization of the myocardial cells changes abruptly, moving from an overall similar orientation in compact myocardium to a different orientation of neighboring molecules in trabeculated myocardium. 

The identification of pairs of defects with a 2D topological charge of *m* = ±½ could have a clinical and pathological significance, namely for a better understanding of non-compacted cardiomyopathies in which the TCI is pathological. One would expect that in these cardiomyopathies, pairs of defects with a 2D topological charge of *m* = ±½ would be found much deeper in the LV myocardium due to prominent trabeculae and deep intertrabecular recesses. Assuming that the future identification of defects would be possible on other 2D cross-sectional imaging such as CT, this could be a tool to aid in the diagnosis of such pathologies.

Interestingly, although we found many pairs of defects with a 2D topological charge of *m* = ±½, those with a 2D topological charge of *m* = +1/2 were the most numerous. In addition, those present near the luminal extremity of the LV trabeculae were mostly isolated defects with a 2D topological charge of *m* = +1/2. The comet-like shape of defects with a 2D topological charge of *m* = +1/2 is an expected geometry at the extremity of trabeculae.

Recently, several articles discussing the biological relevance of topological defects suggest that the comet-like shape of 2D topological charge *m* = +1/2 defects confers them a capacity of self-mobility and propulsion in active matter. Due to their propensity for self-propulsion, they tend to accumulate in regions of low activity and in boundaries, in contrast to stationary defects with a 2D topological charge of *m* = −1/2 [1,15,16]. Please note that these observations were made in single-layer or multi-layer liquid crystals and cannot be simply extrapolated to a 3D nematic liquid crystal-like myocardium. This work has yet to be carried out.

As a reminder, in our previous article [4] we visually identified two types of topological defects within the compact ventricular mass. The former were defects with a 2D topological charge of *m* = −1/2, identified at the anterior and posterior part of the IVS, and the latter defects were those with a 2D topological charge of *m* = +1 located at the LV apex of our specimens. The contribution of the automatic and quantitative analysis of topological defects presented in this article makes it possible to compare these results. 

The compact ventricular wall defects with a 2D topological charge of *m* = −1/2 were visually as well as algorithmically identified at the anterior and posterior part of the IVS. While the vast majority of the defects identified in the trabecular myocardium were present in pairs of the opposite 2D topological charge (*m* = ±½), those located at the anterior and posterior part of the IVS were isolated. As no other topological defect was present, this explains why it was easier to identify them visually. This observation is noteworthy, especially since this zone corresponds to a particular anatomical entity, namely an area where the compact myocardium of the LV and RV are entangled. 

The defects with a 2D topological charge of *m* = +1 visually detected at the apex of the ventricular mass were not automatically detected. This is an understandable limit of the algorithm. These defects correspond to a vortex of the entire ventricular apex. For automatic detection it would be necessary to increase the length of the rotor, but in doing so the rotor would rotate largely outside the ventricular mass, preventing any robust data extraction.

Finally, a major limitation of this study is to give only the mean number of topological defects with the same 2D topological charge *m* per section. This approach has the advantage of simplicity but is imperfect. Indeed, 80 to 90% of short axis sections concern the right and left ventricular cavities, and 10–20%, the left ventricular cavity and the inflow and outflow of the RV. Due to the complexity of cardiac anatomy, robust methods will need to be implemented to measure the possible variation in the number of defects depending on the location of the inflow, the trabeculated chamber, or the outflow of the RV.

## 5. Practical Conclusions

We have shown that it is possible to automatically detect topological defects with a 2D topological charge of *m* = +1/2 and *m* = −1/2. This is possible thanks to the spatial resolution of our data that is of the order of 100 µm. This article explores the significance of this new tool on a series of normal perinatal and infant hearts limited to a 2D space. We hope to present, in future, the results in a 3D space in a collaborative work with physicists. 

Furthermore, this new tool is not specific to the fetal period. Cardiac CT will soon achieve this spatial resolution in vivo. We hope to have made the principles underlying the automatic extraction of singularities sufficiently clear so that the community of clinicians can take advantage of this new technique to explore all the pathologies of the interface between the compact myocardium and the trabeculated myocardium, as well as the changes that take place around areas of the pathological myocardium.

## Data Availability

The data presented in this study are available on request from the corresponding author.

## Supplementary Materials

The following supporting information can be downloaded at: https://www.mdpi.com/article/10.3390/jcdd11010011/s1, Videos S1–S3: Topological defects maps of entire hearts where red crosses represent the centers of defects with a 2D topological charge of *m* = +1/2 and blue crosses represent the centers of defects with a 2D topological charge of *m* = −1/2; Video S1: Topological defects map of a perinatal heart from apex to base; Video S2: Topological defects map of an early infant heart from apex to base; Video S3: Topological defects map of a perinatal heart in transverse plane from anterior to posterior.

## Appendix A. 2D-Topological Charge Calculation

We used the azimuth map images of a section to compute the topological defects map as follows: For each pixel belonging to the ventricular mass, we defined a pivotal center. The calculation of the 2D topological charge implies a process generating a loop that rotates a bar of a given length (i.e., arbitrary radius of 4 pixels) around each pivotal center (one full turn = 2π) and sums up the variation in vector directions observed at the tip of the bar (i.e., the azimuth angle of the closest pixel from the tip). This signed sum is then divided by 2π, and expresses the 2D topological charge or winding number of the defect at this pivotal center (i.e., the current pixel).

Definition: for a pixel (*x_c_*, *y_c_*) the formula of the topological charge m for a given radius *r* is:

$$m=\frac{1}{2\pi }\sum_{\alpha =\varepsilon }^{\alpha =n.\varepsilon }\left[\Phi \left(x_{c}+r.\cos\left(\alpha \right),y_{c}+r.\sin\left(\alpha \right)\right)-\Phi \left(x_{c}+r.\cos\left(\alpha -\varepsilon \right),y_{c}+r.\sin\left(\alpha -\varepsilon \right)\right)\right]$$

where *ɸ* is the azimuth data; *r* the radius; ε *= 2 π/n* and *n* is the number of summation steps.

**Algorithm A1.** Algorithm in pseudocode
let PHI a source image containing the azimuth data and of dimension dimx, dimy
let M a destination image to store the measured topological charges and of dimension dimx, dimy
let epsilon an optimal increment angle for the rotation taking discretization into account
let n the number of summation steps
let rd be the radius
%% select the number of summation steps as a function of the discrete radius
n = floor(rd) * 8
%% select the min threshold value for a significant calculation
min_count = n * 0.9
for row ← 0 to dimy step 1
for col ← 0 to dimx step 1
sum ← 0
count ← 0
xc ← col
yc ← row
xd ← xc + rd
yd ← yc
if (xd,yd) in muscle
azi ← PHI(yd,yd)
sum ← sum + prev_azi - azi
prev_azi ← azi
endif
for alpha ← epsilon to 2*pi step epsilon
ca ← cos(alpha)
sa ← sin(alpha)
xd ← xc + rd * ca
yd ← yc + rd * sa
if (xd,yd) in muscle
azi ← PHI(yd,yd)
sum ← sum + (prev_azi - azi)
count ← count + 1
prev_azi ← azi
endif
endfor
if (count > min_count)
sum ← sum / (count)
sum ← sum /(2 * pi)
M(xc,yc) ← sum
endif
endfor
endfor
